# The role of cities in reducing the cardiovascular impacts of environmental pollution in low- and middle-income countries

**DOI:** 10.1186/s12916-020-1499-y

**Published:** 2020-02-24

**Authors:** Jill Baumgartner, Michael Brauer, Majid Ezzati

**Affiliations:** 1 Institute for Health and Social Policy, McGill University, Montreal, QC Canada; 2 Department of Epidemiology, Biostatistics, and Occupational Health, McGill University, 1110 Pine Avenue West, Montreal, QC H3A 1A3 Canada; 3 Department of Epidemiology and Biostatistics, School of Public Health, Imperial College London, London, UK; 4 School of Population and Public Health, The University of British Columbia, Vancouver, BC Canada; 5 Institute for Health Metrics and Evaluation, University of Washington, Seattle, WA USA; 6 MRC Center for Environment and Health, Imperial College London, London, UK; 7 WHO Collaborating Centre for NCD Surveillance and Epidemiology, Imperial College London, London, UK

**Keywords:** Developing countries, Heavy metals, Household air pollution, Inequalities, Noise, Urban

## Abstract

**Background:**

As low- and middle-income countries urbanize and industrialize, they must also cope with pollution emitted from diverse sources.

**Main text:**

Strong and consistent evidence associates exposure to air pollution and lead with increased risk of cardiovascular disease occurrence and death. Further, increasing evidence, mostly from high-income countries, indicates that exposure to noise and to both high and low temperatures may also increase cardiovascular risk. There is considerably less research on the cardiovascular impacts of environmental conditions in low- and middle-income countries (LMICs), where the levels of pollution are often higher and the types and sources of pollution markedly different from those in higher-income settings. However, as such evidence gathers, actions to reduce exposures to pollution in low- and middle-income countries are warranted, not least because such exposures are very high. Cities, where pollution, populations, and other cardiovascular risk factors are most concentrated, may be best suited to reduce the cardiovascular burden in LMICs by applying environmental standards and policies to mitigate pollution and by implementing interventions that target the most vulnerable. The physical environment of cities can be improved though municipal processes, including infrastructure development, energy and transportation planning, and public health actions. Local regulations can incentivize or inhibit the polluting behaviors of industries and individuals. Environmental monitoring can be combined with public health warning systems and publicly available exposure maps to inform residents of environmental hazards and encourage the adoption of pollution-avoiding behaviors. Targeted individual or neighborhood interventions that identify and treat high-risk populations (e.g., lead mitigation, portable air cleaners, and preventative medications) can also be leveraged in the very near term. Research will play a key role in evaluating whether these approaches achieve their intended benefits, and whether these benefits reach the most vulnerable.

**Conclusion:**

Cities in LMICs can play a defining role in global health and cardiovascular disease prevention in the next several decades, as they are well poised to develop innovative, multisectoral approaches to pollution mitigation, while also protecting the most vulnerable.

## Background

Exposure to environmental pollutants has emerged as an important but significantly understudied risk factor for the development of cardiovascular diseases (CVD) [1], which form the leading cause of death globally. Up to 90% of the estimated global CVD burden is borne by populations in low- and middle-income countries (LMICs), threatening public health and economic development [2]. Air pollution and lead exposures accounted for an estimated 10% of all deaths and 17% of cardiovascular deaths in 2017, as reported by the Global Burden of Disease study. Air pollution alone was responsible for 12 and 17% of all CVD deaths in the rapidly developing countries of China and India, respectively, along with nearly a million premature CVD deaths in these two countries alone [3]. While the proportional CVD risks of pollution are much lower than those of behavioral risk factors (e.g., tobacco use, harmful use of alcohol, and unhealthy diet) [4], the population impacts of pollution are enormous given the number of people exposed [5]. Over 90% of the world’s population lives in places where air pollution surpasses levels stated by World Health Organization (WHO) guidelines [6] and hundreds of millions of people are exposed to high levels of lead and other heavy metals in their food and water [7]. The high prevalence of exposure suggests a large potential for pollution mitigation to reduce CVD.

Cities in LMICs can play a defining role in pollution reduction and global CVD prevention in the next several decades. Worldwide, cities contain 55% of the population, a proportion that is expected to increase to 68% by 2050, with an estimated 83% of the global urban population living in LMICs [8]. The health benefits of living in urban as opposed to rural areas are well documented [9], where urban dwellers benefit from improved access to healthcare and other public services [10]. However, cities also concentrate industry, traffic, and waste at unprecedented scales, which can lead to high levels of population exposures to contaminated soil, water, and air [1, 11]. Further, the management and mitigation of environmental pollution in LMICs is challenged by the widespread diffusion of multiple pollutants from multiple sources and sectors and a lack of technology, financial resources, and protective environmental regulations.

An often cited justification for these environmental trends is that high levels of pollution are a largely inevitable outcome of economic development [12, 13] and urbanization [14], with some evidence of this trend also at lower levels of development. In Accra, Ghana, for example, informal electronic waste recycling employs thousands of residents and plays a pivotal role in the local economy, but is also a source of high exposure to heavy metals (i.e., lead, mercury, cadmium), flame retardants, and other pollutants among workers as well as adults and children living near waste sites [15]. In Dhaka, Bangladesh, tanneries are a priority economic growth sector and leather is a major export [16]. However, wastewater discharged from the hundreds of local tanneries is a major source of air and surface water contamination in the region [17], and of high exposure to heavy metals and other chemicals among workers and local residents [18]. Often historically overlooked in the development–environment equation are the health and welfare impacts of pollution [19], which can themselves impose very large economic and social costs [20]. Conversely, economic development and urban living brings many environmental benefits, including increased access to clean drinking water, improved sanitation, and access to clean household energy.

Spatial patterns of environmental pollution and of the diseases that result from it vary greatly between and within cities, and depend on numerous factors, including population density, land use practices, the location of economic activities, the occupation of urban residents, availability of transportation networks, energy sources, sanitation, and access to healthcare services. It is historically well documented that the urban poor tend to have unsafe housing and poor sanitation, particularly in LMICs. Most recent evidence documents how the urban poor also tend to live closer to industry and other polluting activities, and often bear the highest burden of pollution. Lower-income neighborhoods in Accra are the most densely populated areas and have the highest air pollution [21, 22], an outcome driven by higher levels of household biomass burning, unpaved roads (and thus exposure to dust), and more traffic than in higher-income neighborhoods [23]. Studies from Beijing, China, observed worse air quality in neighborhoods where residents have lower incomes and less education [24, 25], findings that are consistent with studies in North American cities [26, 27]. Residents of the Kibera slums in Nairobi, Kenya, use urban agriculture to improve their food security, but the locally sourced soil used to grow their food is contaminated with heavy metals, including lead, cadmium, and arsenic from nearby industries [28].

In the sections below, we summarize the evidence associating chronic exposure to pollution and the development of CVD, focusing on the most well-studied pollutants, including air pollution, heavy metals, noise, as well as ambient temperature. We identify key knowledge gaps and discuss the unique role of cities in mitigating exposures to pollution and reducing its CVD impacts.

## Main text

### Air pollution

Air pollution is a complex mixture of particulate matter and gases that are emitted from diverse sources, including industry, household solid fuel stoves, motor vehicles, and agriculture. Fine particulate matter < 2.5 μm in diameter (PM_2.5_) can be deeply inhaled into the lungs, and is the air pollutant with the largest population health impacts [29]. Cities in LMICs are most impacted by air pollution due to concentrated poverty, rapid industrialization, a lack of environmental regulations, and often limited enforcement of existing regulations. Urban air pollution in LMIC cities is up to 17 times higher than in Europe and North America [30, 31], and many residents are additionally exposed to indoor and neighborhood air pollution from household solid fuel burning [30, 32]. Exposure to PM_2.5_ from solid fuel stoves is higher than that to outdoor PM_2.5_ in most high-income countries [33], yet there is substantial overlap between indoor (solid fuel) and urban PM_2.5_ exposures in LMICs. In a systematic review, average daily exposures to PM_2.5_ ranged from 40 to 186 μg/m^3^ among solid fuel stove users in Latin America, sub-Saharan Africa, and Asia [33], which overlaps considerably with the outdoor PM_2.5_ levels in the world’s 500 most polluted cities (range: 27–173 μg/m^3^), over 85% of which are in LMICs [30].

Exposure to PM_2.5_ can induce inflammatory and oxidative stress responses, which are underlying mechanisms for CVD and other diseases [29, 34, 35]. Epidemiological and toxicological evidence indicates that PM_2.5_ is causally associated with the development of CVD [35]. Short-term exposure to PM_2.5_ in both high-income countries and LMICs is consistently associated with an increased risk of hospital admissions and incidence of myocardial infarction and stroke [29, 36, 37]. Additionally, although evidence is mostly from high-income countries, long-term exposure to PM_2.5_ over years can increase CVD risk by an even larger magnitude [29, 35]. Even in low PM_2.5_ settings (yearly averages < 9 μg/m^3^), increases in PM_2.5_ are associated with progression in coronary calcification [38], increased risk of ischemic heart disease [39, 40], and CVD mortality [40], indicating that any level of exposure can increase CVD risk.

The impacts and magnitude of long-term exposure in LMICs are less understood. A recent systematic review identified just 17 studies of long-term exposure to outdoor PM_2.5_ and cardiometabolic disease in LMICs [41]. However, most (65%) of these studies were from China and none were conducted in North or sub-Saharan Africa, which represent nearly a fifth of the world’s population. Overall, long-term exposure to PM_2.5_ was positively associated with cardiovascular mortality (effect estimate range: 0.2–6.1% per 10 μg/m^3^) and with CVD-related hospitalizations and emergency room visits (effect estimate range: 0.3–19.6% per 10 μg/m^3^) [41].

Several studies separately evaluated the CVD impacts of solid fuel stove use [42–45]. In China, solid fuel stove use was associated with a greater risk of CVD mortality (range of hazard ratios (HRs): 1.20–1.29) [44]. In Iran, the use of kerosene stoves was positively associated with CVD mortality (HR: 1.11) in adults, though no association was observed for wood-stove users [46]. Most recently, a multi-country cohort study observed an increased risk of CVD hospitalizations, fatal and non-fatal events, and CVD mortality (range of HRs: 1.04–1.10) among users of solid fuel cookstoves [43]. These studies are supported by studies of subclinical CVD endpoints showing higher levels of inflammatory markers, blood pressure, and arterial stiffness in women using solid fuel stoves and with higher PM_2.5_ exposures, with larger associations in older age [42, 47–49]. Similarly, switching from biomass to gas stoves is associated with blood pressure reductions [50].

Nevertheless, the limited epidemiological evidence from LMICs is a significant knowledge gap in understanding the global health benefits of mitigating air pollution in these regions. Whether the exposure–response functions can be generalized to LMICs remains an area of debate [30, 51]. The exposure–response associations in LMICs are likely to be affected by differences in the underlying population health profiles [30], and may also be impacted by differences in the chemical composition of PM_2.5_ from different sources [52–54] as well as co-exposures to other CVD risk factors. Greater evidence from large, prospective studies in LMICs could fill this knowledge gap.

### Lead, cadmium, and arsenic

Epidemiological and experimental evidence across a range of exposures indicate that chronic exposure to heavy metals and metalloids, including lead, arsenic, and cadmium, are associated with CVD development [1, 55, 56], though most evidence is from high-income countries with low-to-moderate levels of exposure. The full extent of heavy metal exposure in LMICs is unknown due to few countries having biomonitoring programs in place, though evidence from individual studies indicates substantially higher exposures than in high-income countries [57–59].

Lead was one of the first pollutants to receive global attention following elucidation of its neuro-cognitive effects in children [58]. Despite remarkable global decreases in blood lead levels following bans on leaded gasoline in many countries [60, 61], pockets of high exposure persist among people living near industry and in areas with less environmental regulation [58, 62, 63]. Studies in high-income countries associate blood lead levels with cardiovascular mortality and clinical outcomes, including coronary heart disease, stroke, and peripheral arterial disease, with associations evident at blood lead levels as low as 5 μg/dL [1, 7, 64, 65]. For reference, an estimated 120 million people had blood lead levels between 5 and 10 μg/dL and approximately the same number had levels > 10 μg/dL in 2000, the majority of whom were living in LMICs [66]. A link between lead and higher blood pressure is reasonably well-established [67, 68], and lead was associated with a reduced heart rate variability and with abnormalities of cardiac structure and function in adults with low exposures in Europe, Korea, and the US [64]. Based on this evidence, lead accounted for an estimated 998,000 cardiovascular deaths in 2017, mostly due to cerebrovascular disease and ischemic heart disease, as well as for 5.6% of the global CVD burden [3].

Cadmium is less researched despite millions of people globally being chronically exposed to high levels of cadmium in their drinking water and food [69]. Among 12 prospective studies in high-income regions with low-to-moderate levels of cadmium, there was supportive evidence of an association with an increased risk of coronary heart disease, stroke, and peripheral arterial disease [70, 71]. No studies have been conducted in LMICs, even though blood and urine levels of cadmium in their general populations can be several orders of magnitude higher than in North America and Europe [59, 72, 73].

Studies on the CVD impacts of cadmium [70, 71] as well as lead [64] in LMICs are limited to a handful of small studies with subclinical outcomes. Blood lead levels were associated with higher blood pressure in adults living in China [74] and Brazil [75], and among industrial workers in Kenya [76]. Higher levels of serum lead and cadmium were correlated with greater carotid-intimal media thickness in a small cross-sectional study of Turkish adults with renal disease [77]. Among those living in a cadmium-contaminated area in Thailand, high exposure to cadmium was associated with dyslipidemia, oxidative stress, and chronic kidney disease [78].

By comparison, the cardiovascular impacts of arsenic exposure in LMICs are better researched [79–81]. Over 100 million people globally are chronically exposed to arsenic levels above 50 μg/L, primarily though drinking water and food crops grown in arsenic-contaminated soil [82]. Systematic reviews identified over 10 studies from high exposure areas in Taiwan, Bangladesh, Chile, China, inner Mongolia, and Pakistan that consistently found associations between high levels of arsenic in drinking water (> 100 μg/L) and CVD mortality (pooled relative risk: 1.32 [79]), ischemic heart disease, and peripheral arterial disease [80]. Cross-sectional studies in highly exposed populations in Taiwan and Bangladesh showed associations between arsenic and hypertension, though an exposure–response study of arsenic and blood pressure in a lower-exposure region of Mexico did not [83]. This latter study reflects the inconsistent evidence from high-income countries associating low-to-moderate exposures to arsenic with CVD outcomes [79–81].

### Noise

Exposure to environmental noise from transportation and other sources (e.g., people, industry) increases with urbanization and urban densification [84], and a growing body of evidence links chronic exposure to noise with a greater risk of CVD [85]. Studies from high-income countries observed associations between exposure to transportation noise (road traffic, aircrafts, railways) with increased risk of CVD and metabolic diseases. Road traffic noise was associated with myocardial infarction in case–control and longitudinal studies, with the associations increasing after excluding participants with hearing impairment [85, 86]. Experimental and panel studies have consistently observed active positive associations between noise and subclinical markers, including blood pressure, heart rate, and the release of stress hormones [85, 87].

As traffic is also a source of air pollution, which is itself a risk factor for CVD, the question of whether the effect of noise was confounded by air pollution was also evaluated. A systematic review on this topic concluded that the correlations between PM_2.5_ and noise were low to moderate (range of correlations: 0.16–0.72) and that confounding of cardiovascular effects by noise or air pollution were small (< 10%) [88]. However, all reviewed studies were conducted in high-income countries. In a large German cohort, long-term exposure to PM_2.5_ and traffic noise were both independently associated with markers of atherosclerosis [89].

There are no studies of noise and CVD in LMICs, where the levels and sources of noise are considerably different from those in higher-income settings [90]. The few available exposure studies in LMICs measured traffic-related noise and indicated relatively high levels of exposure. Average daytime and night-time sound levels were respectively in the range of 51–108 dB and 44–82 dB in urban areas of Ghana, Turkey, India, Pakistan, and Nigeria [91–94]. By comparison, the WHO Environmental Noise Guidelines recommend the maintenance of traffic noise levels below 53 dB and 45 dB in the daytime and nighttime, respectively, as noise above these levels is associated with adverse effects on sleep and health, including CVD [84].

### Ambient temperature

A number of studies have shown associations between increased cardiovascular mortality and both high and low ambient temperature. A recent study of 340 cities and metropolitan areas from 22 countries (9 of which were classified as developing economies) estimated that 0.54% (95% CI: 0.49–0.58%) and 6.05% (95% CI: 5.59–6.36%) of mortality in those cities were respectively attributable to heat and cold [95]. Among the relatively few studies conducted in LMICs, high and low temperatures were associated with increased cardiovascular risk in India [96], many cities across China [97, 98], and multiple countries in Latin America and sub-Saharan Africa [99–103]. In these studies, low temperatures contributed to higher attributable risks of CVD and all-cause mortality than higher temperatures, supporting findings from high-income countries [104]. In South Africa, for example, the estimated attributable mortality was 3.0% for low and 0.4% for high temperatures [99].

Compared with high-income settings, populations in LMICs are more likely to live in homes and environments that do not adequately protect against the heat or cold. The role that housing and other urban characteristics play in modifying the direct effects of temperature on CVD is poorly understood, particularly for LMICs [103], but limited evidence indicates that these factors may impact vulnerability. Seasonal differences in blood pressure were smaller in regions with central heating in a multi-provincial study in China [105], and support randomized trials showing that indoor heating reduces blood pressure [106, 107]. Studies in Europe found associations between excess winter deaths from CVD and poor housing conditions, including lack of central heating and poor insulation [108, 109]. A recent evaluation of 340 cities observed that the effects of heat on mortality were higher in cities with greater inequality, worse air quality, fewer green spaces, and lower availability of health services [95].

There is an urgency to better understand the temperature–CVD relationships in LMICs, and how features of housing and other urban characteristics can modify these associations when considering the near-term opportunities for intervention. Many LMIC cities are undergoing major growth and revitalization of their infrastructure and physical form amidst urban densification and expansion, providing opportunities to develop new regulations and norms for building construction and to implement well-informed policies and government programs to upgrade existing infrastructure and reduce vulnerability to temperature.

### The role of cities in mitigating pollution and related CVD burden

Age-adjusted cardiovascular disease mortality rates have generally been decreasing and are the main driver of decline in the non-communicable disease mortality rates in LMICs, though the pace of decline varies substantially across countries [110]. Given the rather large (14.6%) estimated attributable fractions of CVD burden described above for environmental risks, developing and applying population-wide strategies to mitigate environmental risks in LMIC cities, where pollution and people are most concentrated, could help accelerate this CVD decline.

Historically, environmental pollution has received less attention in global and national health agendas compared with infectious diseases like HIV/AIDS, tuberculosis, and malaria [20]. Cities are central to pollution mitigation efforts because they sit at the interface of local action and of national and international pollution commitments like the UN Sustainable Development Goals. Importantly, cities have already demonstrated a capacity to more readily respond to environmental issues in the face of global and national-level inaction [111]. For decades, the focus on the global response to climate change was on countries, which have proven largely unsuccessful in producing comprehensive agreements or taking action. By contrast, cities around the world have prepared risk assessments, setting air pollution reduction targets, and pledging to act [112].

Cities are sources of innovation for solutions for reducing pollution because they are most directly impacted by its health and economic impacts. The physical environment of cities can be improved though various municipal processes, including urban planning, infrastructure development, energy and transportation planning, and public health. In cities in Brazil and Colombia, implementation of extensive bus rapid transit systems with features including at-level boarding, prepayment, and articulated busses led to reductions in traffic congestion, travel time, and energy consumption at a fraction of the cost of proposed alternatives, including road and highway expansion [113–115]. Beijing introduced rationing policies to reduce traffic congestion, including driving restrictions (i.e., certain vehicles cannot be used at certain times) [116] and a vehicle quota system that restricted the number of license plates allocated to residents [117]. Congestion charging and low emission zones are being considered by officials in Delhi and Beijing and have been implemented in a growing number of cities, including Milan, Stockholm, Singapore, and London, where there is evidence of lowered traffic emissions [118] and improved road safety and journey times [119].

Citywide implementation of air quality and temperature indices combined with public health warning systems can be cost-effectively used to inform residents of poor air quality and weather events (e.g., high or low temperatures, flooding, drought), and to encourage the adoption of pollution- or weather-avoiding behaviors [120, 121]. While most common in North America and Europe, major cities in China and a growing number of cities in India have implemented early-warning systems that notify residents of poor air quality and alert health professionals to prepare for air pollution-related hospitalizations. In 2014, Ahmedabad, India, was the first city in South Asia to implement a heatwave early warning system [122], with preliminary evidence indicating lower summertime mortality rates after its implementation and larger declines at the highest temperatures [123].

Evidence-based clinical approaches can also be leveraged [124]. Healthcare professionals can develop a suite of targeted community-tailored intervention packages for their service populations. Populations with or at risk of CVD and who live in highly polluted cities may benefit from the targeted use of therapies known to prevent CVD events, including antiplatelet agents, statins, and treatments for hypertension and diabetes [125]. The relative effectiveness of these approaches in highly polluted compared with less-polluted areas is unclear, but they are potential short-term public health interventions.

Local regulations can be used to incentivize or inhibit the polluting behaviors of industries and individuals. As part of a multi-sectoral approach to reduce regional air pollution, the Beijing government banned household coal heaters in millions of homes and simultaneously offered large subsidies for electric heaters and electricity [126]. National bans on leaded gasoline substantially reduced lead exposures in many countries (up to 90%), although lead remains a public health concern in several places [60, 62, 63, 127]. Concentrated poverty combined with a deteriorated housing stock contributes to pockets of high exposure to lead paint in US cities; in response, several affected cities introduced new lead prevention policies and programs, including education, housing inspections in high-risk areas, and local ordinances that require abatement for rental properties [128], with some evidence of success [129, 130].

Past experiences with pollution mitigation in high-income countries can inform evidence-based policies and regulations in LMICs (Table 1). Policies on emissions-based air pollution control (e.g., regulations that promote cleaner vehicle technologies, power generation, or industrial processes) have been most effective [131]. Zoning laws that separate people and pollution sources (e.g., low or no emission zones; requiring schools, daycares, or elder care facilities to be certain distances from major roadways) are less common, but can also be effective given the large (up to 70%) decrease in traffic-related PM_2.5_ within 150 m of a freeway [132]. Reducing the infiltration of outdoor pollutants into the indoor environment through improvements in mechanical ventilation or building design can further reduce indoor exposure but cannot address outdoor exposure [131]. By comparison, the air quality benefits of technologies that remove pollutants from ambient air are negligible – cities in China, India, Korea, and the Netherlands experimented with outdoor air filtration units that were ultimately deemed costly, ineffective, and impractical. Green infrastructure (i.e., urban trees, green walls and roofs, and other urban vegetation) has been promoted to improve air quality and beautify landscapes in several cities, including London [133], despite limited and conflicting emprical evidence of a benefit. Urban vegetation may provide a very small and highly localized air quality benefit in very specific settings, but it does not effectively remove pollution and can actually lead to deterioration of air quality under various meteorological and urban planning conditions [134]. Case studies from LMIC cities can further help to inform evidence-based solutions, including those that address sources like agricultural burning and household solid fuel stoves.

**Table 1:** Strategies and technologies to mitigate exposure to air pollution (adapted from Burns et al. [131] and Rajagopalan et al. [35])

**Societal and governmental interventions**	
*Technology and infrastructure*	
• Cleaner vehicles, e.g., lower or no emissions vehicles, investment in public transportation, reduce the sulfur content of motor fuels	
• Less polluting industrial sources and transport, e.g., implementation of emissions filters and better equipment (i.e., diesel particle traps, catalytic converters)	
• More efficient energy generation – lower emission fuels or renewable sources (e.g., wind, solar) for energy generation	
• Increased investments in cycling and public transport infrastructure to encourage active transport	
*Increasing the distance between populations and pollution*	
• Zoning laws, e.g., requiring new residential areas, schools, daycares and elder care facilities to be located at certain distances from major roadways or polluting industries	
• Shifting polluting industry away from city centers, restrict trucks from city centers	
*Education*	
• Air quality monitoring combined with public health warning systems	
• Publicity campaigns to increase public awareness and compliance alongside pollution prevention initiatives	
• Media campaigns to mitigate lobbying activities by industries involved in power and transport	
*Regulation and policy*	
• Congestion charging schemes	
• Residential wood or coal burning bans or regulations	
• Emissions standards	
**Individual or household interventions**	
*Improving indoor air quality*	
• Switch to cleaner-burning fuels for fireplaces and cooking stoves	
• Use of mechanical ventilation and/or reducing use of natural ventilation to reduce infiltration of polluted outdoor air into homes and buildings	
• Installation of portable air cleaners in homes	
*Behavioral change*	
• Avoid commutes during rush hour or avoid busy roads during active transport	
• Transition to healthier lifestyles (e.g., exercise, healthy diet) to reduce risk of comorbid conditions that increase vulnerability to the health impacts of air pollution; preventative medications and screening programs	

Ensuring that new environmental policies and regulations do not simply shift polluting industries into poor communities and further increase urban environmental inequality (Table 2) will require careful monitoring and extensive dialog and consultation between researchers, officials, and other stakeholder groups. On a global scale, lower costs of labor and production in transitioning economies have attracted new industry, leading to higher levels of air and water pollution [140]. Similar trends have occurred within countries; for example, efforts to reduce urban air pollution in China and India by simply transferring polluting industries to peri-urban and rural areas continued to generate regional air pollution and may have worsened exposures for rural populations who are already more vulnerable and have less access to healthcare [141]. In Beijing, poorer homes in regions where a coal ban was implemented had difficulty in shouldering the additional electricity costs and had colder indoor temperatures [126], which are themselves risk factors for higher blood pressure and CVD mortality [105, 142].

**Table 2:** Exporting pollution to low- and middle-income countries (LMICs)

**Higher polluting cars and fuels**	
African cities are urbanizing and motorizing more rapidly than any other continent. Most countries in sub-Saharan Africa import many more used cars than new ones. Nearly all cars imported into Kenya were previously owned, for example, shipped mainly from Japan and Europe, where they may not meet their stringent environmental standards [135]. Used cars offer a more affordable way for residents to become more mobile but also tend to emit higher levels of pollution [136]. Lower quality diesel fuel, common in many LMICs, further increases pollution emissions from these older cars [137].	
**Hazardous waste from electronics**	
LMICs continue to receive a large proportion of global hazardous waste, including used lead-acid batteries and discarded electronics. A portion of this waste is often inappropriately disposed of into uncontrolled landfills and another portion is recycled, often in informal settings that involve rudimentary processing and disposal methods [138]. These practices contaminate the local environment as well as exposure workers and nearby residents to heavy metals, flame retardants, and other pollutants [139].	

## Conclusion

The UN Sustainable Development Goals and the WHO Global Action Plans target a one-third reduction in premature mortality from non-communicable diseases by 2030. There is substantial evidence from high-income countries and growing evidence from LMICs that reducing population exposures to environmental pollution may accelerate progress in decreasing the global burden of CVD and meeting this target. Though rapid industrialization and urbanization has undoubtedly contributed to high levels of exposure in many LMIC settings, particularly in urban areas, these high levels of pollution are not unavoidable outcomes of these shifts. The health and economic benefits of managing and mitigating pollution are increasingly well recognized, and the behavior of prioritizing economic growth over environmental protection is shifting. Rapidly growing cities in LMICs will play a defining role in global health and CVD prevention in the next several decades as they are well poised to develop innovative, multisectoral approaches to pollution mitigation while also protecting the most vulnerable.

## Data Availability

Not applicable.
